# Rapid growth rate responses of terrestrial bacteria to field warming on the Antarctic Peninsula

**DOI:** 10.1038/s41396-023-01536-4

**Published:** 2023-10-23

**Authors:** Alicia M. Purcell, Paul Dijkstra, Bruce A. Hungate, Kelly McMillen, Egbert Schwartz, Natasja van Gestel

**Affiliations:** 1 Department of Biological Sciences, Texas Tech University, Lubbock, TX USA; 2 Center for Ecosystem Science and Society, Northern Arizona University, Flagstaff, AZ USA; 3 Department of Biological Sciences, Northern Arizona University, Flagstaff, AZ USA; 4 TTU Climate Center, Texas Tech University, Lubbock, TX USA

**Keywords:** Soil microbiology, Climate-change ecology, Microbial ecology, Climate-change impacts

## Abstract

Ice-free terrestrial environments of the western Antarctic Peninsula are expanding and subject to colonization by new microorganisms and plants, which control biogeochemical cycling. Measuring growth rates of microbial populations and ecosystem carbon flux is critical for understanding how terrestrial ecosystems in Antarctica will respond to future warming. We implemented a field warming experiment in early (bare soil; +2 °C) and late (peat moss-dominated; +1.2 °C) successional glacier forefield sites on the western Antarctica Peninsula. We used quantitative stable isotope probing with H_2_^18^O using intact cores in situ to determine growth rate responses of bacterial taxa to short-term (1 month) warming. Warming increased the growth rates of bacterial communities at both sites, even doubling the number of taxa exhibiting significant growth at the early site. Growth responses varied among taxa. Despite that warming induced a similar response for bacterial relative growth rates overall, the warming effect on ecosystem carbon fluxes was stronger at the early successional site—likely driven by increased activity of autotrophs which switched the ecosystem from a carbon source to a carbon sink. At the late-successional site, warming caused a significant increase in growth rate of many *Alphaproteobacteria*, but a weaker and opposite gross ecosystem productivity response that decreased the carbon sink—indicating that the carbon flux rates were driven more strongly by the plant communities. Such changes to bacterial growth and ecosystem carbon cycling suggest that the terrestrial Antarctic Peninsula can respond fast to increases in temperature, which can have repercussions for long-term elemental cycling and carbon storage.

## Introduction

The Antarctic Peninsula is warming at a faster pace than the global average [1–3]. Terrestrial ice-free regions on the Antarctic Peninsula are expanding, creating new habitats for microbial, plant, bird, and mammal communities [3, 4]. The cold and nutrient-limited Antarctic environments combined with the continent’s geographic isolation have resulted in a simplified food web, where microorganisms are the key players in biogeochemical cycling [5–7]. Post deglaciation, these ice-free ecosystems are dominated by metabolically diverse microorganisms that cycle nutrients and determine ecosystem carbon loss and gain. Autotrophic microorganisms in these ecosystems are responsible for fixing carbon, fueling growth and activity of the heterotrophic microbial community [8, 9], ultimately providing the environment with nutrients for plants to develop [10–12]. Taxonomic and metabolic diversity of microorganisms in Antarctica have been determined [13–20], yet we lack quantification of activity of these diverse microbial populations. One metric of activity—growth—is an important indicator of the physiology of a microbial taxon and influencer of soil carbon. Knowing which microorganisms are growing, rates of individual microbial growth, and how microorganisms influence the ecosystem under warming will allow a better understanding of these microorganism-dominated ecosystems and how they might change in the future.

Primary succession generally leads to increases in soil organic carbon, changes in plant community composition, changes in microbial biomass, activity, and community composition [21–26]. Recently, deglaciated soils are low in carbon and nitrogen and are dominated by cyanobacteria [27, 28]. In a glacier forefield, microbial biomass and activity increase along the chronosequence [29], with highest values where plants are present [30]. On the Antarctic Peninsula, bryophytes dominate late successional glacier forefield ecosystems due to their resilience and cold tolerance [31]. In particular, two moss species, *Chorisodontium aciphyllum* and *Polytrichum strictum* form extensive peat banks [32–34]. Microorganisms associated with mosses are important for plant health and are critical mediators of the nutrient cycling [31, 35–39]. Mosses in Arctic and boreal ecosystems are responsible for a significant portion of primary production, regulating soil moisture and temperature, nitrogen availability via their bacterial symbionts, and carbon storage [40, 41]. Mosses in high-latitude ecosystems are sensitive to temperature change [42–47]. However, the activity of microorganisms associated with Antarctic bryophytes and their responses to warming are not well understood. As these ecosystems continue to expand and temperatures on the Antarctic peninsula continue to rise, we must understand the role that moss-associated microorganisms play in ecosystem function. Warming on the Antarctic Peninsula has increased moss peat bank growth and accumulation, and future warming is predicted to increase greenness, following similar trends as Arctic ecosystems [44, 48–50]. Moss microbiomes are likely to continue to be key players in nutrient cycling and ecosystem carbon gain and loss as these areas continue to warm, especially in maritime Antarctica where moss peat banks represent the climax community.

Studies aiming to understand how terrestrial Antarctic microbial communities have changed in response to warming and since deglaciation have indicated a variety of microbial responses [19, 44, 51–53]. Cyanobacterial abundance [27] and rates of carbon and nitrogen cycling of microbial communities have increased with warming in Antarctic soils [54]. The microbial community composition along a glacier forefield chronosequence changed substantially [52] and community-level nitrogen fixation and heterotrophic denitrification rates have increased since deglaciation [55]. A 3-year soil warming study on the Antarctic Peninsula indicated changes in microbial community composition and a loss of functional diversity [19] while another 4-year warming study indicated no change in community composition [15]. Microbial community productivity measurements indicated an increase in microbial activity associated with Antarctic mosses since the 1960s in response to climate warming [44, 49]. These microbial community-level assessments are informative—however, microbial communities are complex and individual taxa grow and assimilate nutrients at different rates [56–58]. Because soil microorganisms control the release and storage of carbon [59], growth increases in response to warming could result in greater soil carbon storage especially if death and turnover increase, contributing necromass to the soil carbon pool [60]. Warming can stimulate the growth and productivity of carbon-fixing microbial taxa, directly contributing to biogeochemical cycling, providing nutrients for biomass increase of neighboring microbial populations—also increasing the soil carbon pool. Higher temperatures are likely to stimulate microbial activity in Antarctic ecosystems where microorganisms live below their optimal growth temperature [61]. The magnitude of the growth response to warming of individual taxa or taxonomic groups may indicate how these microorganisms will impact ecosystem function as temperatures continue to rise. We set out to determine how warming impacts in situ growth rates of microbial taxa in the field along with measurements of carbon fluxes, which will help to understand warming impacts on ecosystem carbon cycling.

We used two sites along the chronosequence of the Marr Ice Piedmont glacier in Antarctica to study how warming impacts the growth of microbial populations in the field. Both sites developed on parent material of glacial till and clay of granitic origin (Bockheim 2015) and have the same climate. They are located within 2 km and are similar in elevation; therefore, are identical in potential biota. The major difference between the sites is that the early successional site, located near the terminus of the Marr Ice Piedmont glacier (lat. −64.77, long. −64.05), is two years post deglaciation, whereas the late successional site, located on Litchfield Island (lat. −64.77, long. −64.09) is ~500 years post deglaciation. Reflecting this difference in time since deglaciation, the early successional site has exposed soil with very low organic matter and no plants, whereas the late-successional site is entirely covered by mosses (*Chorisodontium* and *Polytrichum* spp.) over a peat bank. We used open-top chambers [62] to manipulate air and soil temperature which warmed the soil by 2.0 °C at the early successional site and by 1.2 °C at the late successional site. We conducted a quantitative stable isotope probing (qSIP) tracer study with H_2_^18^O to assess how warming impacts the growth rates of individual bacterial taxa. We used qSIP in situ with intact cores to obtain field-relevant bacterial taxon growth rates in Antarctica. The degree to which bacterial taxa incorporate the heavier ^18^O into their DNA when exposed to isotopically enriched H_2_^18^O relative to their natural DNA density (growth in natural abundance H_2_^18^O) is a measure of their growth rate [56, 63, 64]. We compared growth rates of bacteria between the early and late successional sites after 28 days of warming. We also measured rates of ecosystem respiration (ER) and net ecosystem exchange (NEE), which we used to calculate gross ecosystem productivity (GEP) to understand how the ecosystem is responding to warming. In the absence of plants at the early successional site, carbon fluxes are driven solely by microbial activity.

We hypothesized that microbial community composition would be different between the early and late successional sites and that total growth would be greater at the late-successional site compared to the early successional site due to greater biomass and plant interactions at the former. Because many Antarctic soil bacteria are known to be living below their optimal growth temperature [61], we further hypothesized that microbial community growth rate would increase with warming—more so at the late-successional site due to higher substrate availability. The warming effect on microbial growth should thus also increase rates of ecosystem respiration and productivity. Finally, we hypothesized that the growth responses of individual taxa would be variable in magnitude and the majority of taxa would increase their growth rate with warming.

## Materials and methods

### Site description

The Marr Ice Piedmont glacier is located on Anvers Island, behind Palmer Station, West Antarctic Peninsula. The Marr Ice Piedmont glacier has retreated ~500 m from the station since 1960. Our study took place at two sites along a primary productivity gradient within the chronosequence of the Marr Ice Piedmont glacier near Palmer Station (Supplementary Fig. 1). The early successional site (lat. −64.7736, long. −64.0398) is located near the glacier terminus, deglaciated ~2 years prior to our experiment. This recently deglaciated site consists of a rocky terrain and absence of plant cover. The late successional site (lat. −64.77, long. −64.09) is on Litchfield Island in an Antarctic Specially Protected Area (ASPA 113, ACA permit 2019-007) in a densely vegetated moss-dominated climax ecosystem (100% plant cover), and was last deglaciated hundreds of years prior to our experiment [65]. This site has the same parent material as the early successional site and is on the same glacier forefield, the Marr Ice Piedmont [65]. Litchfield Island is an Antarctic specially protected area due to its high diversity in mammal, bird, and plant habitats, and its unique topography, therefore limited science is performed at Litchfield Island.

The annual mean air temperature at Palmer Station between 1975 and 2018 was −1.99 °C ± 1.02 with a steady increase over time (Supplementary Fig. 2). Mean annual precipitation at Palmer Station between 1989 and 2019 was 21.47 ± 6.87 mm per year. Since 1989, annual precipitation at Palmer Station has decreased (Supplementary Fig. 3).

### Soil properties

Total soil carbon and nitrogen, pH, and gravimetric moisture content were measured from the same soils thawed from −80 °C storage for DNA extraction. For gravimetric moisture content, triplicate 1 g of soil per treatment was dried at 105 °C for 24 h. Soil samples from the five ecosystem replicates per site and temperature treatment were weighed and analyzed for total carbon and nitrogen on an isotope ratio mass spectrometer (IRMS) at the Colorado Plateau Stable Isotope Lab at Northern Arizona University. To measure soil pH at the early successional site, 1 g of wet-weight soil from each plot (*n* = 5, for each treatment) was added to 5 ml deionized water. To measure pH of the late successional site moss, only a 1:5 ratio of moss to water was used. After water addition, samples were homogenized and left at room temperature for 30 min prior to measurement with an electrode. Fresh soil samples (Palmer station lab) as well as frozen (NAU lab) were used to measure pH and the average and standard deviation was calculated. Characteristics of the soil environments at these two sites including gravimetric water content, pH, and soil organic matter are reported in Supplementary Table 1.

### Experimental soil warming design

**Fig. 1:**
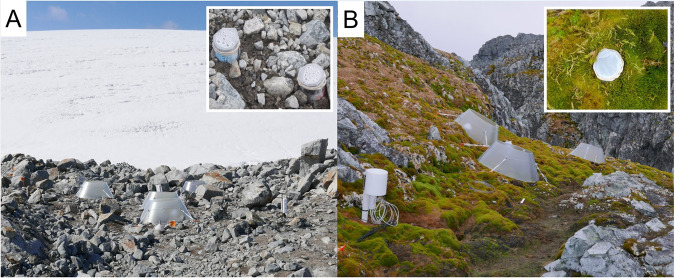
Experimental sites and field tracer study set up. Images of the field qSIP warming study sites, the (**A**) early successional, non-vegetated, recently deglaciated on Anvers Island and the (**B**) late successional, vegetated, on Litchfield Island. The insets represent the experimental cores for the field quantitative stable isotope probing tracer study.

On January 4, 2019, we set up five replicates of 84.6 cm diameter control and open top cone chamber (warmed) plots near the Marr Ice Piedmont Glacier terminus (early succession) and in a *Chorisodontium-Polytrichum* peat bank on Litchfield Island (late succession) (Fig. 1). The open-top chambers had a conical design made of fiberglass material (Sun-Lite HP, Solar Components Corp., Manchester, NH, USA), which have been successfully used in the International Tundra Experiment to passively increase temperatures in high latitude ecosystems by trapping solar energy [62]. At the early successional site, the plots were not on a slope, however, at the late-successional site, the plots were on a north-facing slope, because vegetation on level ground is subject to trampling by seals. The elevation at the early successional site is ~10 m and the late successional site is 25 m.

### Intact core field qSIP tracer study

On January 6, 2019, we used sterile stainless-steel cores (3.5 × 6 cm) with a tapered edge-driven into two random locations in each of the five replicates of the control and warmed plots in the early succession site. At the late succession site, we used a scalpel to cut a cylinder of moss 3 cm in length, fit into the bottom of a sterile sawed-off 50 ml centrifuge tube (core diameter 2.5 cm). This was also conducted for the five replicates of the control and warmed treatments, two qSIP cores for each experimental plot. The early succession cores were carefully removed from the plots, 1 layer of each parafilm and surgical tape, secured by masking tape were placed on the bottom of each core. Then, 5 ml of either natural abundance ^18^O-water (molecular grade Fisher Scientific) or 98 atom percent ^18^O-H_2_O (Isoflex USA, San Francisco, USA) were added to the pair of cores per each experimental plot. The cores were then covered with parafilm, surgical tape, and secured by masking tape and placed back in the plots. At the late succession site, the moss cores in the 50 ml centrifuge tubes received 5 ml of either natural abundance ^18^O-water or 98 atom percent ^18^O water using a double side-port needle, then covered with parafilm, surgical tape, and secured with masking tape, then placed back. This water addition doubled the water content of the samples. However, both glacial melt streams and significant snowmelt occur seasonally in these ecosystems so we are simulating these events in our study. These field qSIP tracer study cores were left in their respective plots in the field for 28 days. After 28 days, the cores were flash frozen, stored at −80 °C at Palmer Station, shipped to Northern Arizona University at −70 °C, then stored at −80 °C. Our study design intended to disturb the ecosystem minimally so we could interpret our results as close to in situ as possible.

During the field qSIP incubation, soil temperature was measured at 5 cm in each plot. The daily mean in the control plots at the early and late successional sites were 6.8 ± 0.7 °C and 6.6 ± 0.3 °C (Supplementary Fig. 4). The warming treatment increased soil temperature by 2.0 ± 0.47 °C for the early successional site and by 1.2 ± 0.47 °C for the late-successional site (Supplementary Fig. 4). Consequently, the number of freeze-thaw cycles declined in the warmed plots (Supplementary Fig. 4).

### DNA extraction

The soil cores were thawed, weighed, and the top 3 cm were removed and homogenized for DNA extraction. A subset of soil was used for obtaining dry mass measurements in a 105 °C drying oven for 24 h. For the moss cores, sterile scissors were used to cut up the moss prior to DNA extraction. DNA from 9 to 10 g wet weight soil from the early successional site and 6–7 g of wet-weight moss from the late-successional site were extracted using the DNeasy PowerMax Soil Kit (Qiagen) following the kit protocol with minor adjustments. To increase DNA yield, the bead tubes with soil and directed solutions were vortexed on max power for 30 min as opposed to the 10 min as written in the manufacturer’s protocol. The entire volume of each supernatant was transferred to the following step and solution C6 was heated to 65 °C before DNA elution. The C6 solution was added to the column 1 ml at a time for a total of 3 ml, with a 5 min wait time between elutions to concentrate the DNA into a smaller volume as opposed to the 5 ml direction of the kit protocol. DNA concentration was measured using a Qubit with PicoGreen (ThermoFisher Scientific, Massachusetts, USA). Some samples resulted in low DNA concentrations that required concentrating prior to ultracentrifugation. For these samples, an isopropanol precipitation with glycogen was used as follows: 200 µl DNA extract + 400 µl DNase free water + 5 µl glycogen (20 mg/mL) + 605 µl 70% isopropanol. The DNA was pelleted at room temperature, centrifuging at 13,400 × *g* for 30 min and cleaned with 500 µl of 70% ethanol.

### Ultracentrifugation and DNA density separation

3 μg of DNA from each sample, 3.675 ml of saturated CsCl (density 1.9 g/ml), and a remainder volume of gradient buffer were added to a 4.7 ml OptiSeal ultracentrifuge tube (Beckman Coulter, Inc USA) and masses of tubes were equalled with gradient buffer. Tubes were capped and inverted 5× and centrifuged in an Optima Max benchtop ultracentrifuge, using a Beckman TLN-100 rotor (Beckman Coulter, Inc USA) at 127,000 × *g* (60,000 rpms) at 18 °C for 72 h. 22–24 DNA density fractions of 200 µl were collected, purified, and quantified as previously described [64].

### Quantitative PCR

qPCR was performed on each SIP fraction and whole DNA (non-fractionated) sample for each experimental replicate using a BioRad CFX-384 thermal cycler as previously described [64]. This totaled 40 whole DNA samples (2 sites × 5 ecosystem replicates × 2 temperature treatments × 2 isotope additions) and 920 SIP fractions (23 SIP fractions × 40 DNA samples). Standards were prepared by amplifying DNA extracted from soil in this study using bacterial (EUB338F/EUB518R) 16S rRNA gene primer sets [66]. This primer set targets solely the bacterial domain, representing 76% of the bacteria contained in the SILVA138 database [67]. Triplicate reactions of 10 µl were quantified using the following reaction mix: 1× Forget-Me-Not (Biotium), 0.2 µM each primer, 1 ng template, and remaining volume of molecular grade water. The following thermal cycling protocol was used for quantifying total bacterial 16 S rRNA gene copies: 95 °C for 2 min and 40 cycles of 95 °C for 5 s, 59 °C for 10 s, 72 °C for 10 s, then a melt curve of 0.5 °C intervals, 30 s each, 55–95 °C to determine product specificity.

### 16S rRNA gene amplicon sequencing

16S rRNA gene libraries were prepared using a two-step PCR amplification method as previously described [64] on each fraction and whole DNA (unfractionated) sample. Sequencing primers used were 515FB/806RB that amplify the V4–V5 region [68–70] and targets solely the bacterial and archaeal domains. This primer set captures 84% of both the bacterial and archaeal domains with 16S rRNA gene sequences contained in the SILVA138 database [67]. All qSIP DNA fractions within the density range 1.65–1.74 g/ml were sequenced, capturing the entire DNA density curve. The DNA in each fraction was cleaned using isopropanol precipitation with glycogen as described above. After normalizing amplicon DNA concentration, they were pooled and sequenced (2 × 150 bp pair-ended chemistry). Sequencing took place on a MiSeq System (Illumina) platform at the Environmental Genetics and Genomics laboratory at Northern Arizona University, Flagstaff, Arizona (nau.edu/enggen).

### Bioinformatics analysis of 16S rRNA gene amplicons

Sequences were imported into QIIME2, demultiplexed, and denoised using DADA2. Taxonomy was assigned using the SILVA 138 database and QIIME2 pretrained classifier. The QIIME2 core diversity metrics pipeline was used to explore and visualize beta diversity measures of the whole community sequenced experimental replicates. A feature table containing sequence reads of all ASVs for each qSIP fraction was exported from QIIME2 and then imported into R for further analysis.

### Determination of taxon-specific ^18^O enrichment

**Fig. 2:**
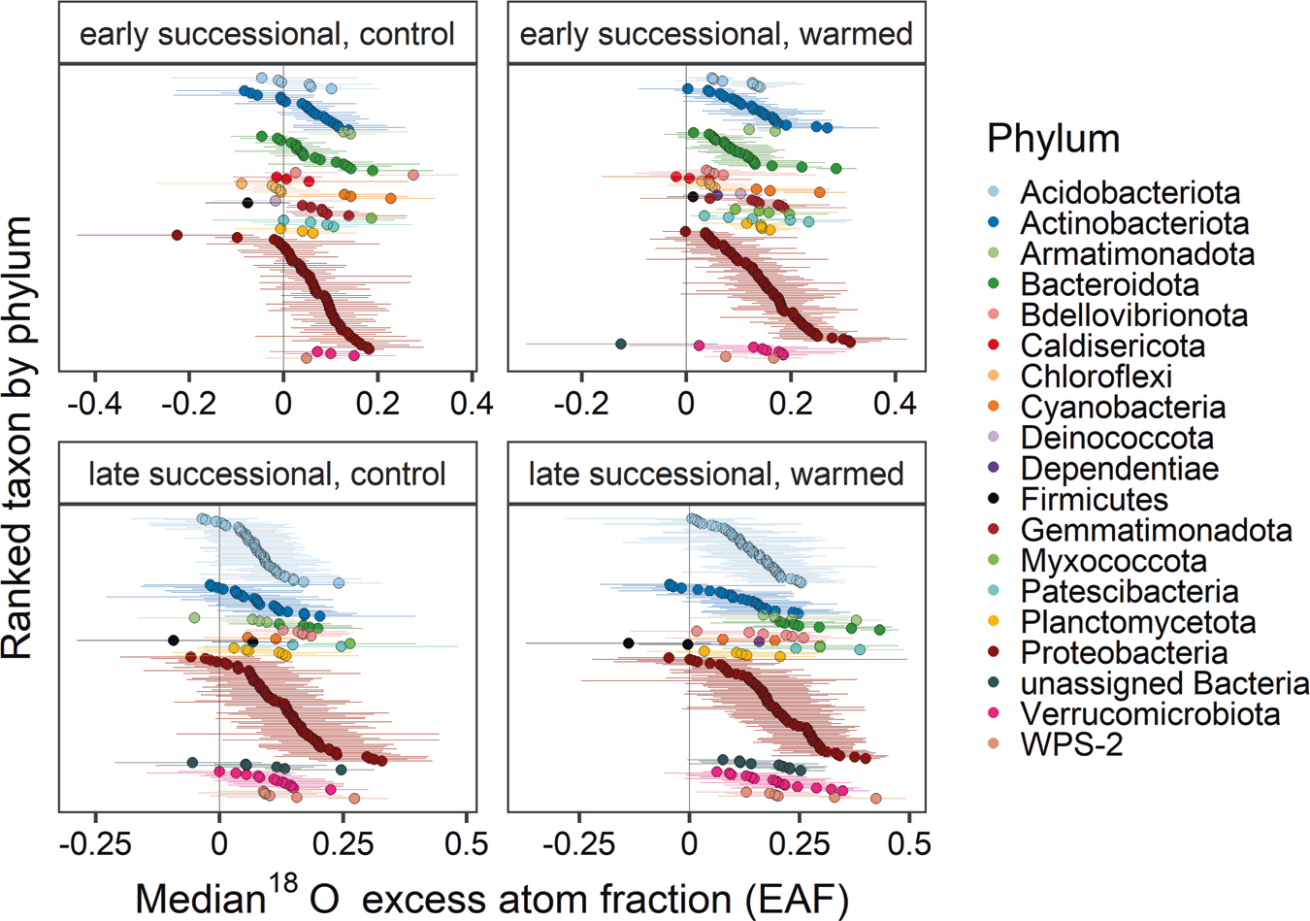
Distribution of taxon ^18^O excess atom fraction (EAF) values for each experimental treatment and after ^18^O enriched water addition in the field. Taxa are colored and ranked by phylum. Points show median EAF and bars show 95% confidence intervals.

Growth rates were determined using quantitative stable isotope probing with ^18^O-H_2_O [56]. The oxygen atoms from this ^18^O isotopically enriched water can exchange with the oxygen atoms in inorganic phosphate resulting in the ^18^O labeling of nitrogenous bases, phosphate, or deoxyribose of actively growing microorganisms [71]. Example calculations and qSIP code to calculate taxon isotopic enrichment have been previously published [56, 63, 64, 72]. Briefly, laboratory data (including density, DNA concentration, bacterial 16S rRNA gene copies for each qSIP fraction), taxonomic metadata, and the QIIME2 exported feature table were all imported into R. Previously developed code was utilized to calculate taxon isotopic enrichment (https://bitbucket.org/QuantitativeSIP/qsip_repo/src/master/) [63]. This calculates the change in weighted average density for each taxon between the two isotope treatments, then the molecular weight change, and ultimately excess atom fraction (EAF), with 95% confidence limits determined using 1000 bootstrapped iterations (Fig. 2). Criteria for including taxa in the analysis included the following: a taxon had to be present in at least four qSIP fractions of two replicates of both natural abundance and heavy ^18^O enriched water treatments for each control or warmed treatment. Relative growth rate (RGR) day^−1^ was calculated as follows: EAF_taxon_/[(average soil water ^18^O enrichment during tracer study)*0.6*28], similar to RGR calculations described previously [57], where EAF is excess atom fraction, 0.6 represents the proportion of oxygen atoms in DNA derived from water [63], and 28 is the length of the field tracer study in days. RGR captures variation among taxa in the proportion of DNA that was newly synthesized during the incubation, and thus is an estimate of relative growth rate for individual microbial taxa. These relative growth rates do not account for taxon turnover during the 28-day incubation, which can result in underestimates of RGR. Our model also assumes populations remained at a steady state, where birth rate and death rate are equal. The average soil water ^18^O enrichment during the tracer study was calculated using a mixing model of the proportion of natural abundance water originally in the sample and the proportion of 98 atom percent enriched ^18^O-H_2_O added to the soil samples. For linear model and phyla differential growth with warming analyses, taxon excess atom fraction and ultimately relative growth rate was calculated per replicate following the published equations [56, 64]. Weighted average density difference of a taxon was calculated for each replicate using the taxon’s average weighted average density across natural abundance oxygen tubes subtracted from each taxon’s weighted average density in each ^18^O tube. Replicate excess atom fraction for each taxon was then used to calculate replicate relative growth rate as described above.

### qSIP statistical analyses

To determine if site, treatment, and their interactive effects were significant in predicting taxon growth rate, we used a generalized linear mixed-effects model. Because of the presence of zeros, we included a zero-inflated structure using glmmTMB [73], where the model was coded as follows: glmmTMB(RGR ~ site * temp_trt + (1 | tube) + (1|ASV), zi = ~1, data = tubelevelRGR, family = gaussian()). This model was selected using AIC model selection, where models differed in GLM families. Total community growth was calculated by multiplying each taxon’s relative growth rate by their 16S rRNA gene copies and summing these values for each treatment. To determine if total community growth differed between the two successional sites and the warming treatment, an analysis of variance was performed on the following model: lm(log(total.growth) ~ site*temp_trt, data = total.growth.tube). The data were log-transformed to fulfill the model assumption that the data are normally distributed. The proportion of cumulative growth attributed to each phylum was determined in each sample. Significant differences between phylum contributions to total community growth were determined also with an analysis of variance using the packages car [74] and emmeans [75] to determine pairwise contrasts between phyla across treatments and the two sites. In R, a linear mixed effects model and variance partitioning analysis was used to determine the variance in bacterial growth rate associated with individual amplicon sequence variant (ASV), warming treatment, and site using the lme4 package [76]. The model was coded as follows for each site data: lmer(RGR ~ 1 + (1|temp_trt) + (1 | ASV), data = pertube.persite. A similar model was used to determine how much variance in relative growth rate was attributed to taxonomy, where taxonomic ranks were nested, coded as follows: lmer(RGR ~ 1 + (1|phylum/class/order/family/genus/species/ASV, data=pertube.persite). The Bray-Curtis, Jaccard, and weighted and unweighted Unifrac distance betadiversity calculations and PerMANOVA analyses were performed in QIIME2 [77]. A principal coordinates analysis (PCoA) of Bray-Curtis distance of relative abundance and relative growth rates was performed in R using the ape package [78]. PerMANOVA (with 9999 iterations) was used to determine if centroids of the four groups (i.e., early and late successional sites, each with control and warmed treatments) differed.

To determine whether taxonomic families increased their relative growth rates with warming within each site, 1000 bootstrapped values of relative growth rate in the warmed plots were subtracted from the relative growth rate measurements in the control plots for each ASV. These bootstrapped values were obtained from the output of the qSIP R function all.taxa.calcs (https://bitbucket.org/QuantitativeSIP/qsip_repo/src/master/) [56, 63]. The 95% confidence intervals of the bootstrapped differences were calculated for each family for which a growth rate was calculated in both the warmed and control plots within a site. A family was considered to have increased its relative growth rate with warming if the 95% confidence interval did not overlap zero.

### Ecosystem carbon flux measurements

We measured ER, GEP, and NEE three times over the course of the 1-month field qSIP in the same plots as the field qSIP (*n* = 5). Both ER and GEP are reported as positive values, but for NEE we used the atmospheric perspective, i.e., negative NEE values indicate a carbon sink. Prior to the start of the measurements, a semi-permanent thin-walled stainless steel soil collar of 30 cm diameter was inserted into the soil (ca. 3–4 cm depth) in the center of each plot soil (i.e., collar heights above the soil surface varied between 2 and 4 cm). The soil collars were used to create a good seal between our flux chamber and the soil. The custom-built flux chamber, made of cast acrylic, had an area of 660 cm^2^ and 8.7 cm height (i.e., total volume of 5.7 l) and contained a fan inside for air mixing. The flux chamber was connected to a LI-6800 (LI-COR, Lincoln, NE) as a closed system.

To measure NEE we recorded the change in CO_2_ concentration for a period of 90 s. To measure ER, we monitored the rise in CO_2_ concentration after blocking light by covering the transparent chamber with a blackout curtain panel. We removed the first 20 s of data collected (i.e., the “deadband”). We determined the slope of the dry CO_2_ concentration (*C*_dry_; mol mol^−1^) over the remaining 70 s. The *C*_dry_ accounts for differences in water vapor concentration and hence standardizes across measurements. The *C*_dry_ was calculated as follows:

1 $${C}_{{{{{{\rm{dry}}}}}}}=\frac{C}{1-\frac{W}{1000}}$$

Where *C* and *W* are the CO_2_ (μmol mol^−1^) and H_2_O (mmol mol^−1^) concentrations, respectively, inside the flux chamber. Using linear regression, we obtained the slope of *C*_dry_ over time, *dC*_dry_/*dt*, which was subsequently used to calculate flux values for NEE and ER as follows:

2 $${flux}=\frac{10{V}_{{tot}}P(1-\frac{{W}_{0}}{1000})}{{ART}}\frac{{{dC}}_{{dry}}}{{dt}}$$

Where *V*_tot_ is the volume of the chamber (cm^3^), adjusted for the height of the soil collar for each plot, *P* is the atmospheric pressure (kPa), *W*_0_ is the water vapor concentration at time 0 (when slope calculations start after the deadband period), *A* is the footprint of the flux chamber (here, 660.5 cm^2^), *R* is the gas constant (8.31 J K^−1^ mol^−1^) and *T* is the temperature (K). To calculate GEP we first negated NEE before adding the corresponding ER values for each plot.

Flux time series data were plotted in R using the package ggplot2 [79]. We performed linear mixed effects model in the package lme4 [76], using plot id and day as random effects to account for the repeated measures in each plot. For fixed effects, we used the main effects of treatment and site, and their interaction. Data were log-transformed to meet the assumption of normally distributed residuals. Because NEE values could be negative, thus precluding log transformations, we shifted values upward such that the minimum value in the data set was 1, thereby enabling log transformation. We used the package lmerTest [80] to get significance values for the fixed effects.

While we generally used an alpha level of 0.05, we opted to discuss results when alpha level was 0.1, because of the high variability in this ecosystem.

## Results

### Bacterial community composition and abundance

ASV richness was higher in both the control and warmed plots of the late site (compared to the early site (Table 1). Similarly, bacterial abundance was about one order of magnitude higher at the late successional site compared to the early successional site (Table 1). Warming for 28 days had no effect on the abundance of bacterial 16S rRNA gene copies at either site (Table 1). (Archaeal 16S rRNA gene sequences were too low in abundance to pass filtering requirements in our sequencing data.) Early and late succession bacterial communities differed in beta diversity (PerMANOVA, *p* < 0.001) but warming had no effect (Supplementary Fig. 5; Supplementary Table 2).

**Table 1:** Richness of taxa present in 16S rRNA gene sequence data, taxa that passed filtering thresholds to calculate a qSIP growth rate for, significantly growing taxa (i.e., 95% C.I. of excess atom fraction value do not cross zero), and bacterial 16S rRNA gene copies per dry gram mass for both sites and treatments.

Successional site and treatment	Total ASVs across fractions	ASVs where we calculated a qSIP growth rate (% community relative abundance)	ASVs significantly growing (% community relative abundance)	Bacterial 16S rRNA gene copies (g dry mass^−1^) (NS between treatments within each site, *p* < 0.001 between sites)
Early Control	1318	131 (91.1 ± 4)	57 (42.4 ± 7.5)	2.52 × 10^9^ ± 8.14×10^8^
Early Warmed	1873	180 (94.9 ± 3)	140 (84 ± 5.9)	2.67 × 10^9^ ± 4.14×10^8^
Late Control	2699	187 (94.3 ± 5)	89 (63.3 ± 5.8)	1.18 × 10^10^ ± 5×10^9^
Late Warmed	2676	210 (95.3 ± 5.2)	153 (88.8 ± 8.8)	1.2 × 10^10^ ± 4.45×10^9^

The number in parenthesis represents the percent relative abundance in that treatment. *NS* = Not significant *p* value from student’s *t* test.

### Bacterial community growth and response to warming

Out of all ASVs present at the two sites and treatments, an average of 9% passed filtering criteria for the qSIP growth rate calculation, representing 91–95% of the community relative abundance (Table 1). ^18^O EAF values were calculated for a total of 708 taxa across all four site by treatment combinations (Fig. 2). For the control plots at the early and late successional sites, ASVs that represented 42% and 63% of the community relative abundance were considered significantly growing ASVs (Table 1). These percentages increased under warming, where significantly growing taxa represented 84% and 89% of the community relative abundance in the early and late successional sites, respectively (Table 1).

**Fig. 3:**
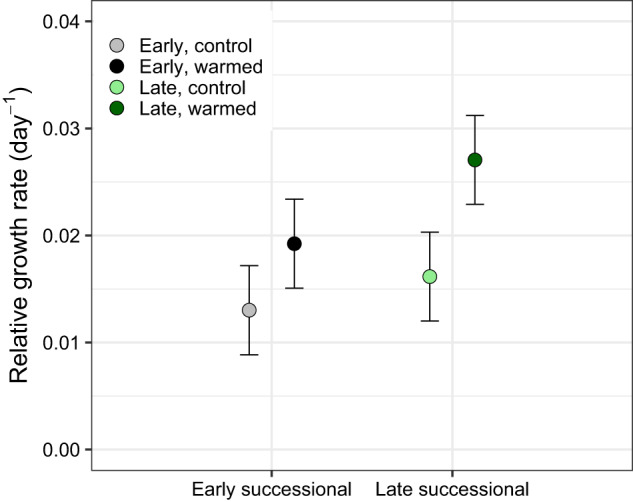
Mean relative growth rates day ^−1^ of all microbial taxa across the early and late successional sites in both the control and warmed treatments. The *p* value indicates significance of the warming treatment determined by Type II Wald Chi-square Test is 0.039. Site had no significant effect on relative growth rate (*p* = 0.191) nor did the interaction between site and temperature (*p* = 0.569).

The mean relative growth rate for the control plots at the early successional site was 0.013 ± 0.004 and was 0.016 ± 0.004 day^−1^ at the late successional site (Fig. 3). The mean relative growth rate for the warmed plots in the early successional site was 0.019 ± 0.004 and was 0.027 ± 0.004 day^−1^ at the late successional site (Fig. 3). Warming had a significant effect on mean relative growth rate across both sites (Type II Wald Chi-square Test *p* = 0.039; Fig. 3). Relative growth rate was not significantly different between the early and late successional sites (Fig. 3). The interaction between site and temperature treatment in predicting relative growth rate was also not significant (Fig. 3).

### Total and phylum contributions to community growth under warming

**Fig. 4:**
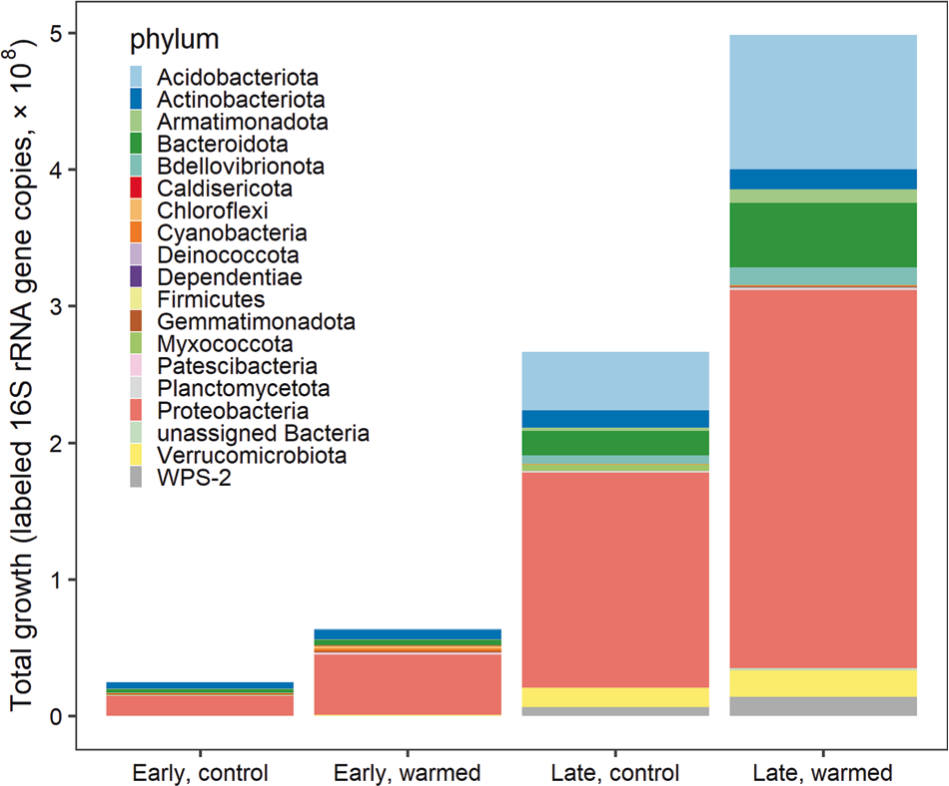
Cumulative growth (RGR × 16S rRNA gene copies per g dry mass per taxon per day) of all taxa in control and warmed plots at both the “early” successional, non-vegetated, recently deglaciated site and the “late” successional, vegetated site at Litchfield Island. Cumulative growth significantly differed between the sites (*p* value < 0.001) and between the control and warming treatments (*p* value < 0.01), significance determined from an analysis of variance type 1 test.

**Fig. 5:**
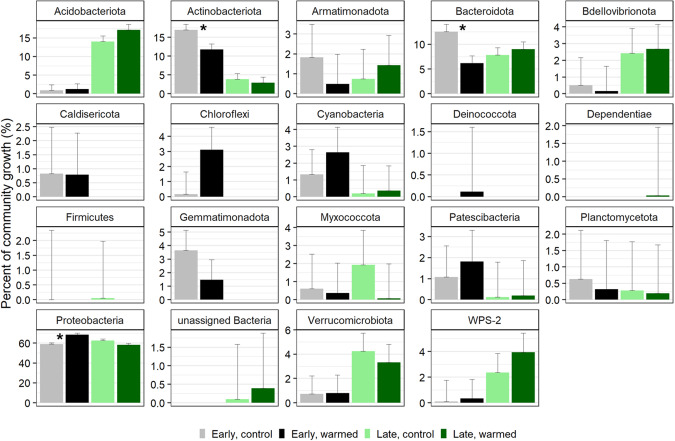
The proportion of total community growth attributed at the phylum level for the early successional, non-vegetated site in control (gray) and warmed (black) plots and the late-successional, vegetated site in control (light green) and warmed (dark green) plots with standard error bars. Asterisks represent the three phyla in the early successional site that had significantly different contributions to total community growth between the control and warmed treatments.

Cumulative growth in the control plots of the early successional site was 2.48 × 10^7^ 16 S rRNA gene copies per gram dry soil per day and was 6.39 × 10^7^ in the warmed plots (Fig. 4). Cumulative growth was higher at the late successional site, as total abundances were higher, totaling 2.66 × 10^8^ in the control plots and 4.99 × 10^8^ gene copies per gram dry mass per day in the warmed plots (Fig. 4). Total growth differed significantly between sites (type I ANOVA, *p* < 0.001) and between the control and warmed treatments (*p* < 0.01) (Fig. 4). Phylum contributions to total community growth ranged from <1 to 68%, where *Proteobacteria* contributed to over 55% of total growth across sites and treatments (Fig. 5). *Proteobacteria* were the dominant phyla at both sites. *Acidobacteriota* (16%) and *Bacteroidota* (8%) had the next highest contributions to growth in the late-successional site and *Actinobacteria* (14%) and *Bacteroidota* (10%) had the next highest contributions to community growth in the early successional site (Fig. 5). Three phyla significantly changed their contribution to community growth in response to warming in the early successional site (Fig. 5). These included the *Proteobacteria* phylum which increased in contribution to community growth and the *Actinobacteriota* and *Bacteroidota* phyla which decreased their contributions to growth with warming (Fig. 5). The relative contribution of each phylum to community growth was not affected by warming at the late-successional site.

### Bacterial taxonomy is important for predicting relative growth rates

**Fig. 6:**
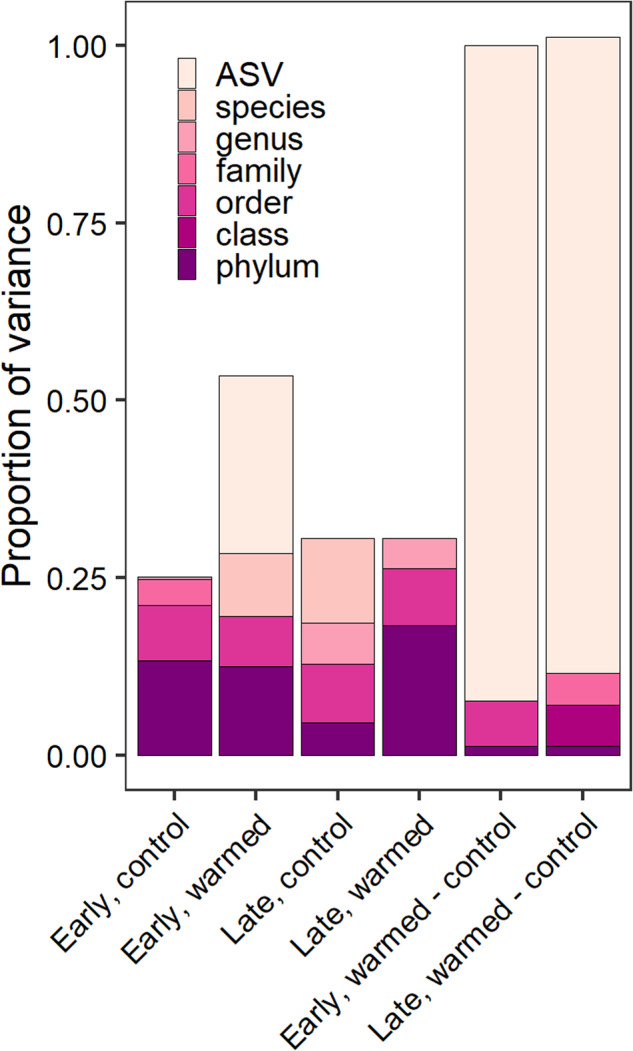
Variance in growth rate explained by taxonomy. The proportion of variance of relative growth rate explained by taxonomic rank for each site and treatment including differential effects due to warming shown to the right.

The highest amount of variance in predicting bacterial relative growth rate was attributed to ASVs for both the early (27.6%) and late (18.4%) successional sites (Table 2). Followed by the warming treatment which explained 17% and 14.5% of the variance in relative growth rate for the early and late successional sites, respectively. More than 50% of the variance was unexplained at both sites (Table 2). On average, taxonomy accounted for 35% of the explained variation in bacterial relative growth rate. Across treatments, the phylum level explained 4.5–18% of the variation in bacterial growth rate and the remainder of variation (12–41%) was explained by the class to ASV level (Fig. 6).

**Table 2:** Variance partitioning from a linear mixed effects model to determine variance associated with the warming treatment and individual ASVs in predicting relative growth rate for the early successional and the late-successional sites.

Model component	Early successional (% variance explained)	Late successional (% variance explained)
ASV identity	27.6	18.4
Treatment	17	14.5
ASV identity × treatment	2.6	0
Unexplained	52.8	67.1

### Many bacterial families increased their relative growth rates with warming

**Fig. 7:**
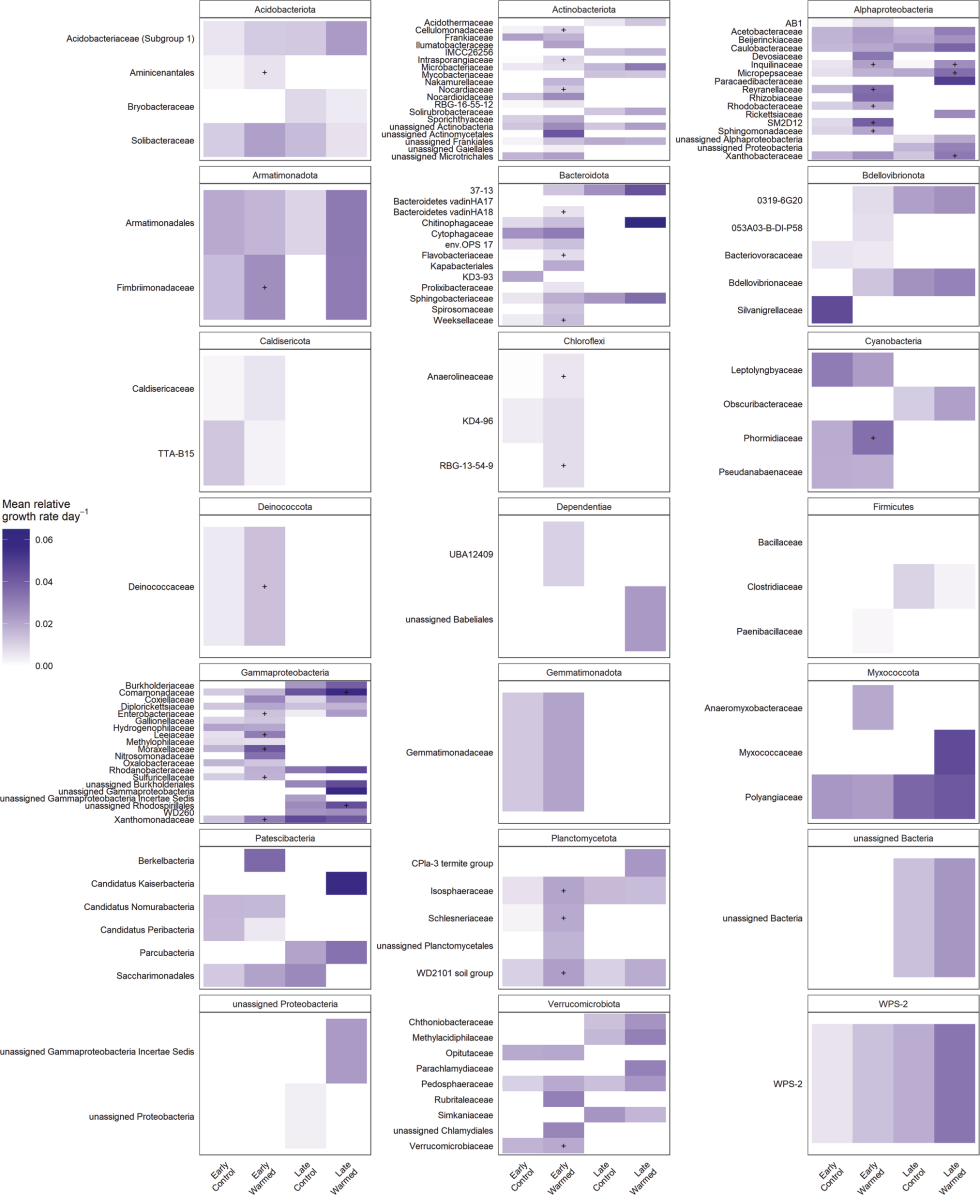
Heatmap of mean relative growth rate per day for taxonomic families grouped by phylum for each of the early and late successional sites and the control and warmed plots. Gray boxes indicate that a growth rate was not calculated for that family due to absence. The “+” indicates a significant increase in growth rate with warming for that family.

Only three ASVs were shared between the recently deglaciated, early successional and the late-successional moss-dominated site. However, many taxonomic families were shared and growing across both sites and treatments including *Acidobacteriaceae* (Subgroup 1), *Solibacteraceae*, *Sphingobacteriaceae*, *Polyangiaceae*, *Isosphaeraceae*, *Pedosphaeraceae*, *WPS-2*, and many families in the *Alphaproteobacteria* and *Gammaproteobacteria* classes (Fig. 7).

There were 26 families in the early successional sites that significantly increased their relative growth rate with warming including *Moraxellaceae*, *Leeiaceae*, and *Reyranellaceae*. Autotrophic families were also responding to warming at the early successional site, including *Phormidiaceae* (*Cyanobacteria*) and *Sulfuricellaceae* (a sulfur-oxidizing chemolithoautotrophic family in *Gammaproteobacteria*) (Fig. 7). There were only five families in the late-successional site that significantly increased their relative growth rate with warming including *Inquilinaceae*, *Xanthobacteraceae*, and *Coxiellaceae* (Fig. 7).

### Responses of ecosystem carbon fluxes to warming

**Fig. 8:**
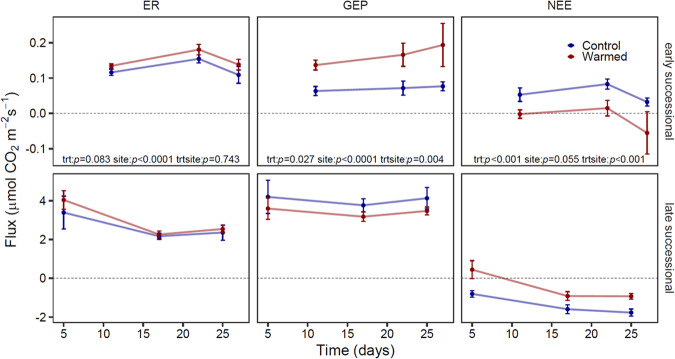
Ecosystem carbon flux measurements over the course of the 28-day field qSIP tracer study at two successional sites along the chronosequence of the Marr Ice Piedmont glacier for both the control and warmed plots. ER ecosystem respiration, GEP Gross ecosystem productivity, NEE Net ecosystem exchange. GEP represents autotrophic activity. Positive NEE represents a carbon source to the atmosphere and negative NEE represents a carbon sink to the ecosystem. Error bars represent the standard error of the mean.

During the 28-day field qSIP study, both sites varied in their ecosystem response to warming (Fig. 8). Overall warming increased ER by 12%, but the effect was only marginally significant (*p* = 0.083). The non-significant site x warming interaction indicated that the direction of the effect was positive for both sites, but rates of ER were lower at the early successional site (0.14 μmol CO_2_ m^−2^ s^−2^) relative to the late-successional site (2.79 μmol CO_2_ m^−2^ s^−2^). Warming stimulated GEP at the early successional site (234%), but decreased GEP (by 15.4%) at the late-successional site (Fig. 7, site × warming interaction, *p* = 0.004). GEP was much lower at the early successional site (0.12 μmol CO_2_ m^−2^ s^−2^ on average) compared to the well-developed late successional site (3.72 μmol CO_2_ m^−2^ s^−2^ on average; effect of site, *p* < 0.001). The effect of warming on NEE depended strongly on site (site × warming interaction, *p* < 0.001): warming caused the early successional site to shift from a carbon source to a carbon sink, whereas warming caused the late-successional site to be a weaker carbon sink (Fig. 8).

### Linking changes to ecosystem carbon fluxes to bacterial responses

Carbon fluxes were relatively more altered by warming at the early successional site than at the late successional site. Carbon fluxes at this recently deglaciated site are solely regulated by microbial activity. The largest changes at this site were that warming increased GEP to such an extent (relative to increased respiration) that NEE became negative (i.e., became a carbon sink rather than a carbon source). This coincided with an increase in the relative growth rates of photoautotrophs (e.g., *Cyanobacteria*) and chemolithoautotrophs (e.g., *Sulfuricellaceae*) (Fig. 7).

## Discussion

Our field incubation study using intact soil cores at warmed and control plots in Antarctica resulted in field-relevant growth rates of microbial assemblages at high taxonomic resolution. We found that soil microbial populations had a strong response to short-term warming at both the early and late successional sites along the Marr Ice Piedmont glacier forefield.

### Bacteria responded to warming by increasing their growth rate

The lack of change in biomass and an increase in relative growth rate indicate increased turnover of the microbial community, consistent with previous findings that soil warming increases microbial turnover [81]. This increased turnover can have consequences for soil carbon, including increases in soil respiration [81] as well as future successional changes to the microbial community [82]. An increase in microbial taxon growth rates of short-term warmed Alaskan soil was attributed to temperature dependent carbon mineralization [83]. In the long term, warming has increased growth rates of bacterial taxa in Alaska and decreased them in a temperate meadow in Arizona [83, 84]. Temporal changes in response to warming can be drastic and non-representative of short-term responses [85], making it imperative to study both long-term and short-term responses. We hypothesize that increased carbon inputs from both carbon-fixing plants and microorganisms in Antarctica over the chronosequence succession and with warming may elicit a future microbial growth response similar to Alaskan microbial ecosystems, where labile carbon inputs from plants likely sustained increased microbial growth rates, counter to a decrease in growth rates and carbon over the long term in temperate ecosystems [84, 85].

Our study indicated that warming increased bacterial growth rates and respiration at both sites. While we expected the vegetated site to have a greater response to warming due to high substrate availability, growth and respiration could have been constrained by other factors including the low nitrogen availability or the acidic physical environment (Supplementary Table 1). Low carbon availability at the early successional site likely attenuated an increase in ER with warming; however increased activity of carbon-fixing microorganisms that we observed in the short-term (Fig. 7) may indicate a more robust ER response in the future.

Warming significantly increased growth rates of individual taxa across both study sites. However, we would not have detected a warming response of the bacterial community if we had relied on community composition data alone (Supplementary Fig. 5; Supplementary Table 2). Previous results of community composition changes with warming have been mixed. In a laboratory incubation, a change in glacier forefield microbial community composition was observed after 40 days of incubation at 5–15 °C [86]. Three years of field warming caused changes in community relative abundance and increased bacterial abundance in high vegetation cover plots [19], yet four years of warming found no changes [15]. In the Arctic, microbial community and biomass changes were only detected after fifteen years of warming [87]. Interpretations from studies assessing relative abundance and concurrent changes in biomass are problematic due to inaccuracies in true abundance changes. Rather than relying on these measurements, we show that qSIP is a more sensitive tool to assess of microbial response in the short term.

Our field warming treatment not only increased the soil temperature, but also decreased the number of days with freezing temperatures (Supplementary Fig. 4). Decreased frequency in freeze-thaw cycles could cause a decline in nutrient availability since freeze-thaw generally increases dissolved organic carbon and nitrogen [88]. The decline in freeze-thaw likely influenced microbial activity in our study, which we interpret as part of the effect of warming.

### Higher total growth in the late-successional site, but not relative growth

Our experiment used space-for-time substitution to infer effects of succession on ecological processes, and as such there is the potential that differences between sites unrelated to time since deglaciation contributed to the effects we observed. This includes any microclimate differences due to the late-successional site’s north-facing slope [33]—although the diurnal thermal profiles of the two sites were similar (Supplementary Fig. 4). Nevertheless, the large differences in plant matter and organic matter accumulation are evident for the entire area around the late-successional site—a direct result of our space-for-time design—which is likely the dominant driver of differences in the microbial community between sites.

While relative growth rate did not significantly differ between the early and late successional sites, total growth was significantly higher at the late-successional site compared to the early successional site, and warmed plots compared to control plots. Bacterial 16S rRNA gene copy abundance and total community growth for the late successional site was higher than early successional site (Fig. 4; Table 1). This is consistent with total community growth from vegetated sites yielding greater growth [61] and higher microbial abundance compared to non-vegetated sites on the Antarctic Peninsula [18]. A meta-analysis across 64 studies found that microbial abundance increased the most under experimental warming in cold, histosol soils which are high in organic matter [89]. Further, moss-microbe symbioses mediate carbon and nitrogen biogeochemical cycling, processes which increase nutrient availability and promote increases in microbial abundance [90]. While we did not detect an increase in bacterial abundance in our short-term warming study, increased growth rates at both sites and higher bacterial abundance and total growth increase at the late-successional site may indicate future changes in biomass and activity as these ecosystems continue to warm. At the late successional, vegetated site, a larger majority of the total community was already growing in the control treatment (63%) and warming stimulated growth for 25% more of the community (Table 1), indicating the late-successional community likely had fewer environmental limitations of growth prior to warming compared to the early successional site. Microorganisms at the early successional site may not only be limited by temperature, but also limited by nutrients such as carbon, nitrogen, and/or phosphorus [91]. These nutrient limitations could be alleviated in the future as deglaciated ecosystems on the peninsula expand and bird and mammal colony habitats form in these systems, influencing the nutrient availability [92]. As early successional sites continue to develop, these results highlight the need for long-term warming studies in these ice-free Antarctic Peninsula ecosystems.

While the mean growth rate increased similarly between successional sites (Table 1), the degree of warming was less at the late-successional site (Supplemental Fig. 4). This suggests that we may expect an even greater temperature response to growth at the late-successional site if the magnitude of warming was as great as the early successional site. The increases in growth at both sites are particularly striking because of the short time span of warming (28 days). If these responses continue to occur over longer time spans, this suggests that future climate warming scenarios will have a dramatic effect on ecosystem function.

Individual microbial taxa and taxonomic groups varied in their relative growth rates and in their response to warming. This suggests that the immediate release from temperature limitation was important and growth rates of microbial populations were influenced by their individual traits. Taxonomic variation in growth rates was larger than the changes in growth caused by warming, which further supports the idea that growth is not solely affected by temperature, but other abiotic or biotic variables (e.g., nutrient availability or physiological traits). In both sites and temperature treatments, taxonomy was an important indicator of relative growth rates. Sometimes at the phylum level and other times at higher taxonomic resolution, explaining 50% of more of the variance in growth rate (Fig. 6). We suspect that phylogenetic relatedness may contribute to microbial growth rate responses as has been shown across multiple ecosystems [93, 94].

### Bacterial phyla growth contributions to community growth under warming

In the early successional site, the *Actinobacteriota* and *Bacteroidota* phyla significantly decreased their contribution to community growth under warming (Fig. 5). *Actinobacteriota* has been found to dominate in Antarctic glacier forefield ecosystems where their presence was correlated with a range of trace elements and salts, indicating a broad metabolism that allows them to colonize and develop soils along glacier forefields [52]. Despite a significant increase in growth of three families in the *Actinobacteriota* phylum, a decrease in growth of other *Actinobacteriota* resulted in overall lowering their contribution to community growth. This suggests a potential shift in actinobacteriotal contribution to ecosystem function. Similarly, *Bacteroidota* decreased their contribution to community growth by about half at the early successional site. *Bacteroidota* have been shown to positively correlate with samples high in C and N [95]. And in Antarctica, they can even shift to a mixotrophic lifestyle when resources are limited where they oxidize hydrogen potentially as a means for survival [20, 96]. We find, however, that under warming in this low-nutrient environment, *Bacteriodota* are likely outcompeted by other taxa that increase their total growth at a faster rate under warming.

### Bacterial families respond positively to warming

Between the early and late successional sites, only one shared family (*Inquilinaceae* of class *Alphaproteobacteria*) had a significantly increased growth rate with warming, an unsurprising result due to the contrasting productivity, characteristics, and successional stage of these two sites. The majority of families that significantly increased growth rates with warming were heterotrophic (e.g., *Cellulomonadaceae*, *Xanthomonadaceae*, and *Sphingomonadaceae*) (Fig. 7). However, at the early successional site in particular, autotrophic families were also responding. Increased autotrophic activity with warming supports the observed increase in gross ecosystem productivity, direct evidence of how increased microbial growth can impact ecosystem carbon cycling at the early successional site. While we did not analyze the growth responses of eukaryotic algae that are likely to be present, these autotrophic taxa are likely contributing to sustaining the heterotrophic growing community under warming in this carbon-limited ecosystem while also contributing to the observed carbon flux response.

### Detecting short-term warming responses of microbial growth

Our study provides the first field in situ growth rates of soil microorganisms responding to short-term warming from a glacier forefield chronosequence and productivity gradient on the Antarctic Peninsula. While we only captured taxa that replicated within 28 days, we likely missed rare taxa with much longer doubling times [97]. However, our qSIP approach was able to calculate growth rates for taxa that accounted for 91–95% of the relative abundance of the total community (Table 1). Under our experimental warming, at least 84–89% of the total bacterial community members replicated during the 28-day incubation indicating we captured the growth of a large majority of the community. At the early successional site, warming stimulated the growth of nearly half of the total bacterial community that otherwise were replicating slowly or not at all. This indicates a substantial change in bacterial activity with a 2 °C increase.

Our analysis did not include fungi or eukaryotic algae, although they are present in these environments [98] and their activity will impact biogeochemical cycling especially due to their higher per capita carbon assimilation rates. Fungi have also been shown to be more sensitive than bacteria to changes in the number of freeze-thaw cycles [51]. The knowledge of the fungal and algal growth response to temperature will be critical to understand the microbial response to warming across these two sites and to determine the effect of microbial communities on carbon and nutrient cycling, especially since evidence suggests increased temperatures may impede fungal growth [99].

## Conclusions

Our study was a short-term 28-day passive warming experiment. This study and future studies that include a longer duration of warming and quantifying the functional changes of the microbial community will allow us to understand how individual microorganisms in these systems will respond as temperatures continue to increase and how these microorganisms impact their environments. Our study shows that bacteria in both early and late succession Antarctic glacier chronosequence sites were able to quickly respond to warming by individually modifying their growth rates. In the absence of plants at the early successional site, we show a direct link between microbial growth, GEP, and NEE, where warming increased microbial autotrophic activity that resulted in the young ecosystem switching from a carbon source to a carbon sink. If this persists long-term, the system could develop quicker with an increased rate of ecosystem carbon storage.

Overall, taxa varied in their growth response, but many increased their growth with warming in both ecosystems. Warming stimulated the growth of taxa that were not growing in the control plots, doubling the number of actively growing community members. The total growth and the total growth response to warming was higher at the late succession vegetated site, indicating that these moss-associated microorganisms under future ecosystem warming scenarios will be critical components of the ecosystem to monitor, especially as moss peat banks continue to expand in Antarctica. These moss-associated bacteria have the capacity to impact carbon cycling where they can turn over quickly with increasing temperatures. Perhaps the more muted response of ER and GEP to warming despite changes to bacterial growth rates at this site is more reflective of the plant community responses. If so, it remains to be seen how the interaction of plants and microorganisms will be altered in the future and whether mature sites become weaker carbon sinks with warming.

The warming response of soil microorganisms across many ecosystems resulted in concurrent increases in growth and soil respiration [100]. Further quantification of individual microbial taxon activity will allow us to determine their contributions to both growth and respiration [101], however changes in autotrophic activity along this chronosequence appeared to drive much of the ecosystem response to warming. As ice-free regions in the Antarctic Peninsula continue to expand, thereby allowing successional changes of the soil biota, understanding the growth and activity of the resident microbial community is critical to determine how these systems will respond to temperature increases and impact nutrient and ecosystem carbon cycling.

### Supplementary information

Supplemental Figures and Tables

## Data Availability

The 16S rRNA gene sequencing data used in this study can be found on NCBI GenBank SRA database under the accession PRJNA906184. The qSIP data produced in this study are available from the corresponding author upon request. The code and functions for analysis used in this study can be found at: https://bitbucket.org/QuantitativeSIP/qsip_repo/src/master/.
