# Supplementary material for: Rapid growth rate responses of terrestrial bacteria to field warming on the Antarctic Peninsula
Source: ISME J. 2023 Oct 23;17(12):2290–302. doi: 10.1038/s41396-023-01536-4 (PMC10689830; doi:10.1038/s41396-023-01536-4)
Supplement: Supplementary file 1 — Supplemental Figures and Tables [file 41396_2023_1536_MOESM1_ESM.docx]

**
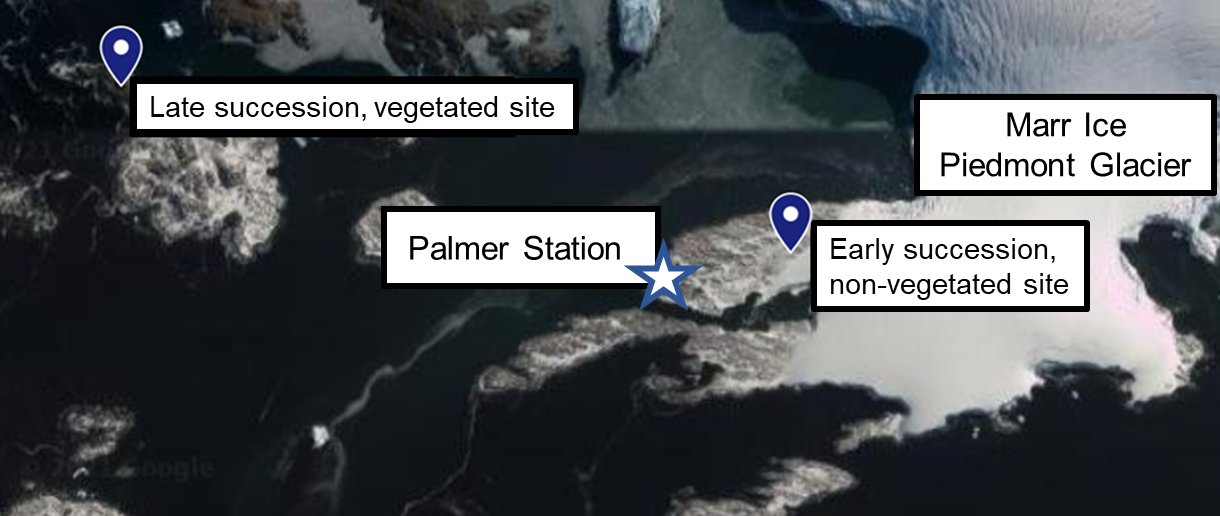
Supplemental Figures and Tables**

**
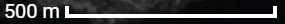
**

Supplemental Figure 1. Map of Palmer Station area showing “early” and “late” successional sites along the chronosequence of the Marr Ice Piedmont Glacier where our field qSIP tracer study in warming plots was performed.


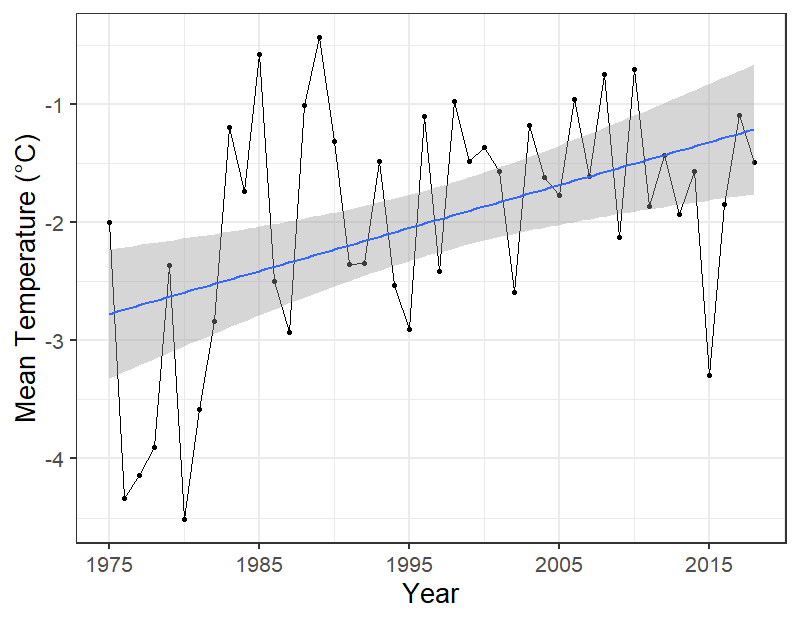


Supplemental Figure 2. Mean annual air temperature at Palmer Station 1975 – 2018. The best-fit regression line is shown in blue and the shaded area represents the standard error bounds of the regression line. Data obtained from annual monthly air temperature data deposited in the LTER network Environmental Data Initiative (EDI) Portal. <https://portal.edirepository.org/nis/home.jsp>


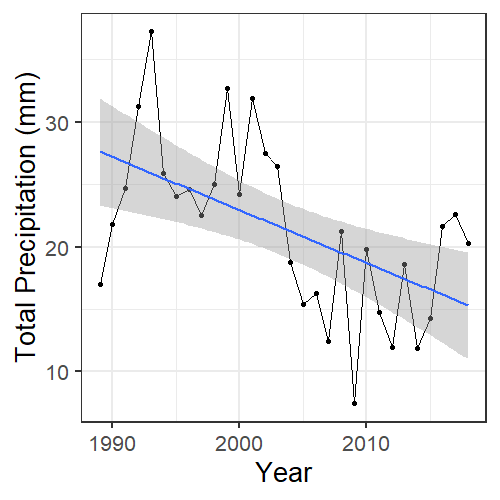


Supplemental Figure 3. Mean annual total melted precipitation at Palmer Station 1989 – 2018. The best-fit regression line is shown in blue and the shaded area represents the standard error bounds of the regression line. Data obtained from monthly precipitation data deposited in the LTER network Environmental Data Initiative (EDI) Portal. <https://portal.edirepository.org/nis/home.jsp>


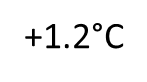

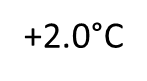

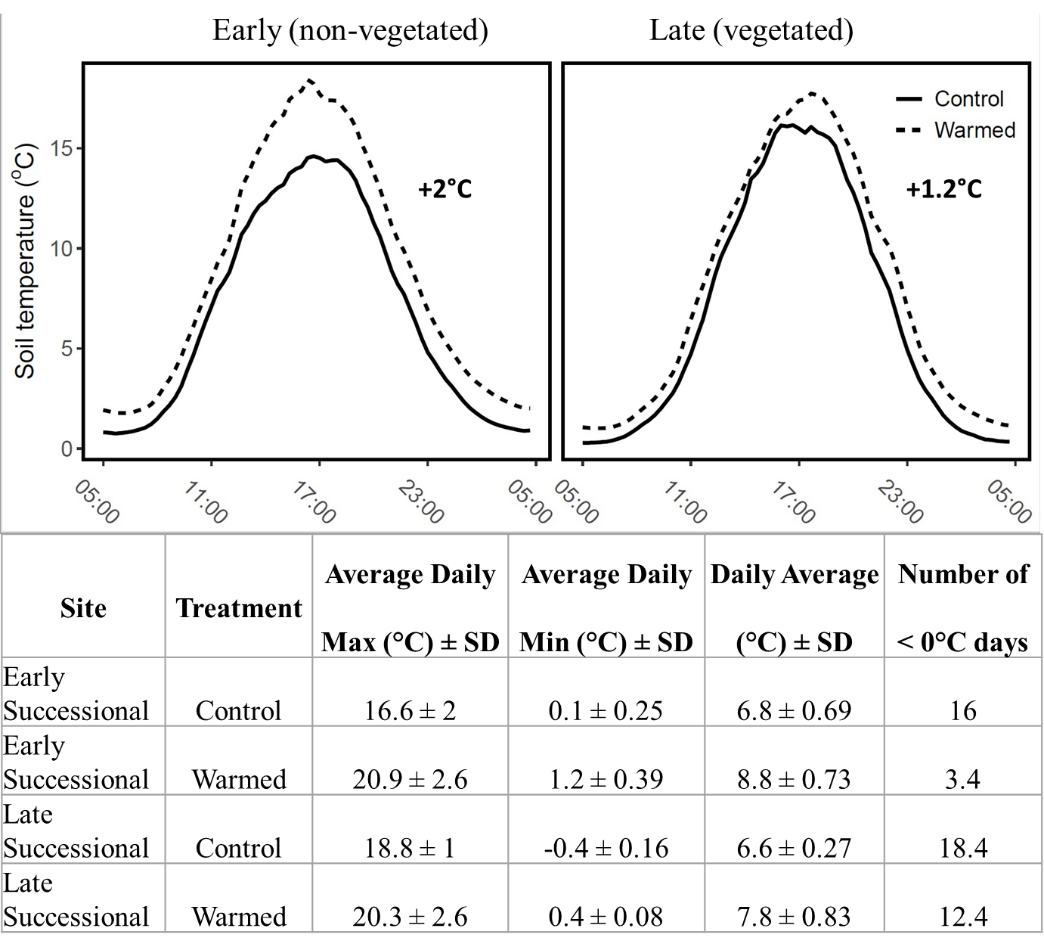


Supplemental Figure 4. Mean soil temperature (measured at 5 cm) throughout the course of a day

during the 28-day field qSIP tracer study at two sites on the Antarctic Peninsula in the control and warmed plots. This includes mean, minimum, maximum soil temperature in each site and treatment with standard deviation as well as number of incubation days where freezing temperatures occurred.


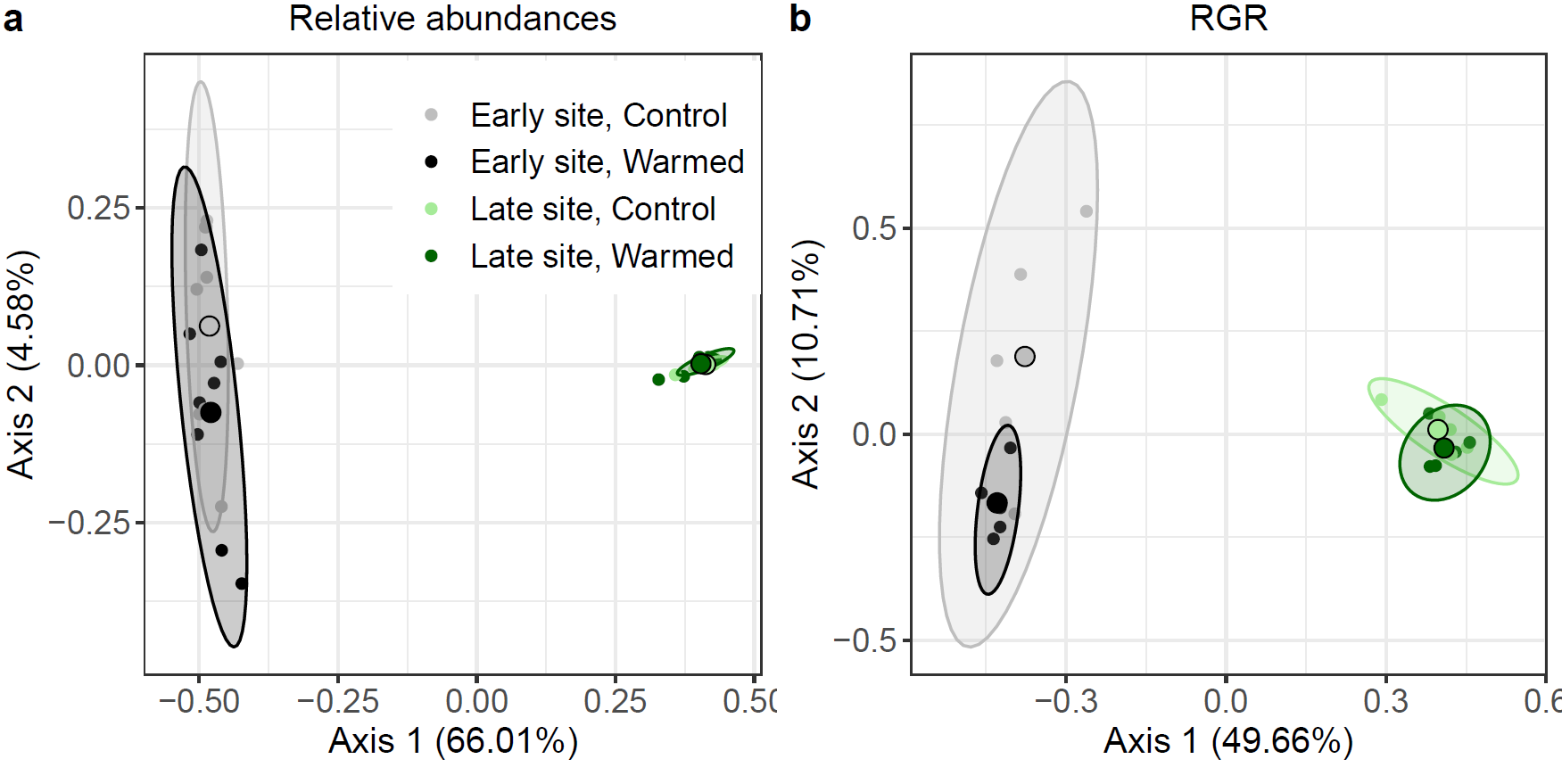


Supplemental Figure 5. Principal Coordinates Analysis (PCoA) of Bray-Curtis distances of relative abundances (a) and relative growth rates (RGR) (b) based on 16S rRNA genes in warmed and control plots at the early and late succession sites. PERMANOVA (with 9999 iterations) was used to determine if centroids (larger circles) of the four groups differed. Results showed that 1) successional stage explained more of the total variation in relative abundances (65.8% of total variation) than relative growth rates (49.0% of total variation) of taxa within the bacterial communities, and hence, axis 1 is representative of successional stage, and 2) that treatment explained very little of total variation (<1%, *P* = 0.32) in relative abundances, but explained more of RGR (5.7%, *P* = 0.05). There was also a weak, but significant treatment x site interaction (*P* = 0.05) for the RGR, but not for relative abundances.

Supplemental Table 1. Characteristics of the soil environment at the early, non-vegetated and late, vegetated sites, values plus or minus standard deviation.

Supplemental Table 2. Beta diversity metric p values obtained from PerMANOVA analysis to compare bacterial community composition between the control and warmed plots at each site after 28 days of warming. (NS) means nonsignificant p value.

| Comparison | Unweighted unifrac | Weighted unifrac | Bray-Curtis dissimilarity | Jaccard |
| --- | --- | --- | --- | --- |
| “Early” site Control vs Warmed | 0.555 (NS) | .11 (NS) | 0.546 (NS) | 0.629 (NS) |
| “Late” site Control vs Warmed | 0.04 (*) | 0.56 (NS) | 0.340 (NS) | 0.428 (NS) |

| Site | Gravimetric moisture content (%) prior to water addition | Soil pH | Total carbon (%) | Total nitrogen (%) | Dominant plant species |
| --- | --- | --- | --- | --- | --- |
| Early (non-vegetated) | 14.6 ± 3.3 | 5.57 ± 0.48 | 0.79 ± 0.07 | 0.07 ± 0.01 | NA |
| Late (vegetated) | 323.3 ± 15.9 | 4.64 ± 0.20 | 43.81 ± 0.61 | 0.69 ± 0.10 | *Chorisodontium aciphyllum*  *Polytrichum strictum* |
